# Mechanical properties of sol–gel derived SiO_2_ nanotubes

**DOI:** 10.3762/bjnano.5.191

**Published:** 2014-10-20

**Authors:** Boris Polyakov, Mikk Antsov, Sergei Vlassov, Leonid M Dorogin, Mikk Vahtrus, Roberts Zabels, Sven Lange, Rünno Lõhmus

**Affiliations:** 1 Institute of Solid State Physics, University of Latvia, Kengaraga st. 8, LV-1063, Riga, Latvia; 2 Institute of Physics, University of Tartu, Ravila 14c, 50412, Tartu, Estonia; 3 Estonian Nanotechnology Competence Centre, Ravila 14c, 50412, Tartu, Estonia; 4 ITMO University, Kronverkskiy pr., 49, 197101, Saint Petersburg, Russia

**Keywords:** atomic force microscopy (AFM), nanomechanical tests, scanning electron microscopy (SEM), silica nanotubes

## Abstract

The mechanical properties of thick-walled SiO_2_ nanotubes (NTs) prepared by a sol–gel method while using Ag nanowires (NWs) as templates were measured by using different methods. In situ scanning electron microscopy (SEM) cantilever beam bending tests were carried out by using a nanomanipulator equipped with a force sensor in order to investigate plasticity and flexural response of NTs. Nanoindentation and three point bending tests of NTs were performed by atomic force microscopy (AFM) under ambient conditions. Half-suspended and three-point bending tests were processed in the framework of linear elasticity theory. Finite element method simulations were used to extract Young’s modulus values from the nanoindentation data. Finally, the Young’s moduli of SiO_2_ NTs measured by different methods were compared and discussed.

## Introduction

Hybrid silica core–shell and empty-shell nanomaterials were intensively investigated in recent time [1]. The sol–gel technology for the synthesis of silica nanomaterials is well established, flexible and cost-effective [2–3]. One-dimensional silica nanostructures have plenty of potential applications due to their optical and chemical properties. These include the chemical protection of environmentally sensitive materials [4–5], biological and biosensing applications [6–8], waveguide optics and photonics [9–12]. However, only a few publications were dedicated to the investigation of the mechanical properties of one-dimensional silica nanostructures, and even less to the ones prepared by sol–gel synthesis. Dikin et al. and Ni et al. studied SiO_2_ nanowires (NWs) grown at high temperature with the vapor–liquid–solid method, by using resonance and the three-point bending methods, respectively [13–14]. Houmadi et al. investigated the mechanical properties of SiO_2_ nanotubes (NTs), which were prepared by sol–gel synthesis using organic NT templates, by using three point bending [15]. The differences of the values of the Young’s modulus measured by the listed methods were approximately 40%, which can probably be attributed to peculiarities of the measurement techniques. The effect of the experimental technique on the measured values of the Young’s modulus was demonstrated by Rohlig et al. for ZnO NWs by comparing the resonant technique, nanoindentation, bending of bridges, and tensile and compressive strain tests [16].

In the case of SiO_2_ NTs it is also important to consider structural peculiarities of the material itself. Silicon dioxide in the form of quartz as well as amorphous silica, is a compound with covalent bonds, which at room temperature is rather brittle and does not allow plastic deformation. In studies dedicated to the mechanical characterization of SiO_2_ NTs and NWs, the material was treated as purely elastic without any plastic yield. However, in recent years plastic deformation of nanomaterials with covalent bonds was demonstrated and investigated by several research teams [17–19]. For instance, when thermally produced silica NWs are irradiated by a moderately intense electron beam (e-beam, 10^−2^ A/cm^2^) in transmission electron microscope (TEM), radiation defects can be induced enabling significant plastic deformations in tensile tests, as was shown by Zheng et al. [20].

In this work, we compared several different nanomechanical testing methods applied to thick-walled SiO_2_ NTs in order to get a deeper insight into the mechanical properties of this promising material. First, in situ SEM cantilever beam bending tests were carried out on half-suspended SiO_2_ NTs. Then AFM was used to perform nanoindentation and three point bending tests. Analytical solutions based on elasticity theory were used to process cantilever-beam and three-point-bending tests data, while the data from nanoindentation experiments were fitted by using finite element method (FEM) simulations and compared with the analytical models (thin shell or membrane model and Hertz model). The problem of indentation of thick-walled elastic NTs was addressed and discussed. To the best of our knowledge, no in situ SEM bending tests, as well as AFM nanoindentation experiments were performed on sol–gel silica NTs previously.

## Experimental

Ag/SiO_2_ core–shell NWs were synthesized by coating Ag NWs (diameter 60–140 nm, Blue Nano) with SiO_2_ by using a well-established sol–gel method [5–6,21]. According to the synthesis procedure silica NTs are expected to be amorphous [5,22]. The empty silica shells (SiO_2_ NTs) were obtained by etching the silver core with nitric acid. Silica shells were deposited from solution on an AFM calibration grating (TGXYZ03, Mikromasch), dried and then washed with deionized water.

First, half-suspended NTs were bent inside a high resolution SEM (HRSEM) FEI Helios Nanolab by using a polar coordinate manipulator (MM3A-EM, Kleindiek) without force registration to study the general flexural behavior of SiO_2_ NTs. Standard contact AFM cantilevers (ATEC-CONT) were used as the sharp probes. No special procedures were needed for fastening the NTs to the substrate. The static friction between the NT and the substrate was high enough to keep the adhered part of the NT in place during the bending.

Cantilever beam bending technique [23–24] was applied to half-suspended NTs inside a TESCAN Vega-II SBU SEM equipped with a *x*,*y*,*z*-nanomanipulator (SLC-1720-S, SmarAct) and a force sensor. The force sensor was made by gluing an AFM cantilever with a sharp tip (ATEC-CONT cantilevers, Nanosensors, *C* = 0.2 N/m) to one prong of a commercially available quartz tuning fork (QTF, ELFA). The tip of the ATEC-CONT cantilevers is tilted about 15 degrees relative to the cantilever, which makes the tip visible from the top. In experiments the QTF was driven electrically by an AC voltage at its resonance frequency in amplitude modulation regime. The signal was enhanced by a lock-in amplifier (SR830, Stanford Research Systems) and recorded together with the data of the displacement sensors of the nanomanipulator. The measurements of the Young’s modulus consisted of a one-directional in-plane bending of half-suspended NT. In all experiments the tip oscillated parallel to the surface of the sample (shear mode) and normal to the NTs. The amplitude signal of the QTF (proportional to the applied force) and the sequence of SEM images of the gradually bent NT were recorded simultaneously during the experiment. More details including the QTF calibration procedure can be found in Supporting Information File 1 or in our previous work [24]. After the experiment the sample was studied in HRSEM in order to determine outer and inner diameter of every measured SiO_2_ NTs.

Nanoindentation and three-point bending tests were done by AFM (Dimension Edge, Bruker) under ambient conditions by using tapping mode cantilevers (PPP-NCH, Nanosensors). The built-in software force–distance spectroscopy routine was used both for nanoindentation and three-point bending tests. Radius of the AFM tip, as well as outer and inner diameters of SiO_2_ NTs were measured in HRSEM.

## Theory

**Figure 1:**
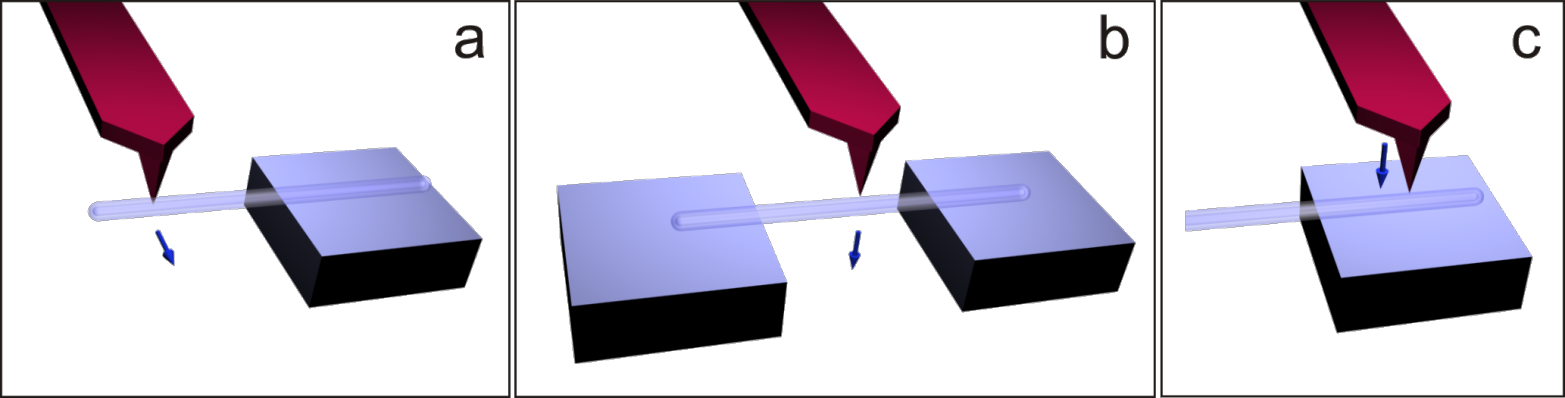
Schematics of mechanical tests performed on SiO_2_ NTs. Cantilever (half-suspended) beam bending inside SEM by using a nanomanipulator equipped with a QTF force sensor (a). Three-point beam bending (b) and nanoindentation (c) by using ambient AFM. The arrows indicate the direction of force loading.

Cantilever beam bending, three-point beam bending and nanoindentation tests were employed to measure the Young’s modulus of SiO_2_ NTs (Figure 1). Each method required different theoretical approaches for analysis and is described in more detail in the following sections.

### Cantilever beam bending

The Young’s modulus of an elastically bent NT can be obtained by applying the equation for the equilibrium of a bent elastic beam with Young’s modulus *E* and area moment of inertia *I* loaded by a point force *F* at its end. The area moment of inertia *I* for a cylindrical shell with outer radius *R*_S_ and inner radius *R*_C_ is given as 
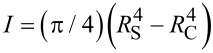
. The force–displacement dependence that accounts for the elastic bending and the tensile strain of an isotropic material can be expressed [25]:

[1]
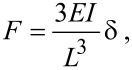


where *L* is the length of the beam, δ is the displacement of the NT end. The knowledge of the geometry of the NT and its force–displacement response is sufficient for determining Young modulus:

[2]
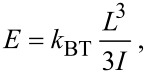


where *k*_BT_ = *F*/δ is the stiffness of the beam measured during the bending test.

### Three-point beam bending

The elastic beam theory is commonly applied for the analysis of the three-point bending tests in the elastic regime [26]. The force–displacement dependence that accounts for the elastic bending and the tensile strain of an isotropic material can be expressed as

[3]
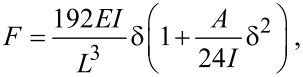


where δ is the displacement of the middle point and *A* is the area of the cross section of the beam. For a cylindrical shell with an outer radius of *R*_S_ and an inner radius of *R*_C_ one gets 
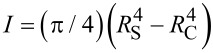
 and 
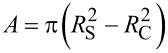
, respectively.

In the linear regime at small displacements the expression for the Young’s modulus can be reduced to:

[4]
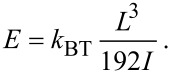


We decoupled raw AFM *F*–δ curves data into the corresponding deformation of the cantilever and of the beam by using the previously measured sensitivity of the cantilever. It enabled us to find the stiffness of the beam, *k*_BT_, and calculate the Young’s modulus with Equation 4.

### Nanoindentation

The analysis of nanoindentation test is more complicated and lacks analytical solutions. The existing model for NT indentation is limited to the thin-shell or membrane case [27]. Therefore, for the case of thick shells studied in this work, we employed finite element method (FEM, COMSOL Multiphysics) models instead, where all geometric parameters can be taken into account. The *Solid Mechanics* module was used, where the thick shell NTs on a SiO_2_ plane were indented with a spherical Si tip. All geometrical parameters of each individual shell studied experimentally were used to build the geometry for each separate FEM simulation. The model used the elastic parameters of the silicon tip: Young’s modulus *E*_1_ = 160 GPa and Poisson ratio *ν*_1_ = 0.22; and the shell: Young modulus *E*_2_ is to be found and Poisson *ν*_2_ = 0.17 [28]. For a reference we have also applied the thin-shell and Hertz models. The thin-shell approach allows one to neglect the indentation of the surface and the tip geometry by taking into account only the membrane-like compression of the shell. The force–displacement relationship for the thin-shell approximation is commonly written as follows:

[5]
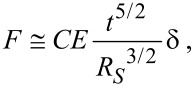


where thickness of the shell *t* = *R*_S_ − *R*_C_ and *C* is a prefactor that depends on the particular boundary conditions with the typical value of *C* = 1.2 [22]. Equation 5 is only applicable within 1% error over the range 0.002 < *t*/*R* < 0.1.

On the contrary, the Hertz model [29] describes only the tip indentation and does not take into account possible membrane-like deformation of the shell. A sphere on a half-space is governed by the following force–displacement relationship:

[6]
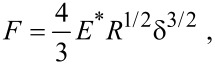


where 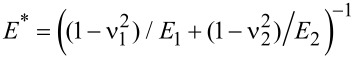 is the reduced Young’s modulus and *R* is the effective tip radius. The Young’s modulus *E*_1_ of the shells found from the Hertz model is underestimated since the shell allows additional elastic relief.

## Results and Discussion

Silica NTs deposited from a solution were randomly distributed over the substrate surface, and many NTs were suspended between neighboring substrate structures, with some NTs being half-suspended (Supporting Information File 1, Figure S1). The NTs appeared semitransparent in the SEM images (at 10–15 kV), which enabled the measurement of both outer and inner diameters of the NTs.

**Figure 2:**
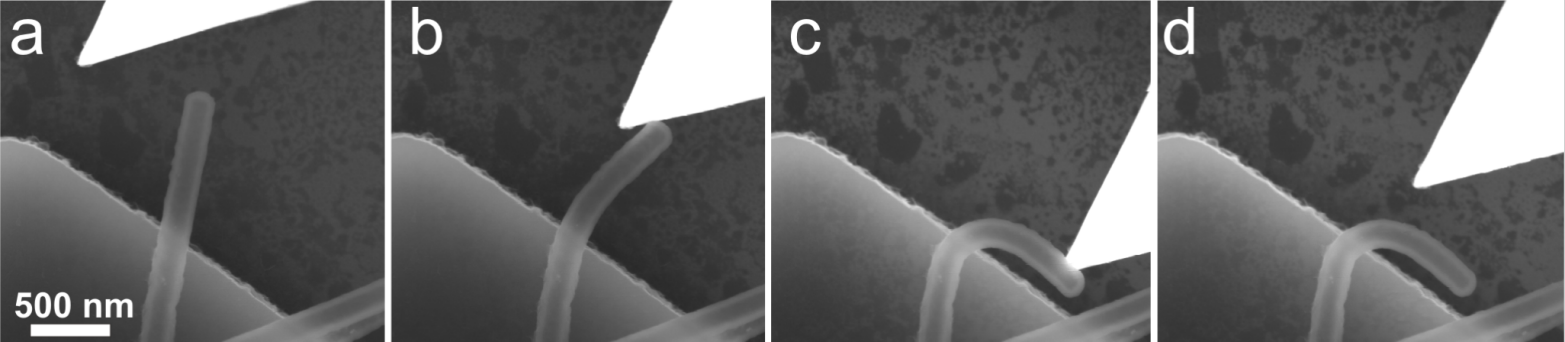
HRSEM images of in situ bending of silica NT. Intact NT (a), slightly bend NT (b), significantly bend NT (c), NT after tip removal (d). Radius of curvature 225 nm.

Bending tests inside the HRSEM revealed a limited elasticity and high resistance to fracture of the half-suspended SiO_2_ NTs. A typical bending experiment is shown in Figure 2. The tip approaches the NT and pushes it near its end (Figure 2a,b). Only negligible elastic shape restoration was observed after tip retraction (Figure 2c,d). Typically, no fracture was observed even at large bending angles (more than 90°). In very few cases, the NTs collapsed during bending (Supporting Information File 1, Figure S2). However fracture was often observed for NTs suspended between two surfaces and pushed in the center.

Cantilever beam bending measurements with force registration were performed on 12 NTs. (A typical measurement is shown in Supporting Information File 1, Figure S3; the results are summarized in Supporting Information File 1, Table S1.) The value of the Young’s modulus was numerically fitted to the experimental force–displacement curve registered by the QTF by using Equation 2. The average value of the Young’s modulus was 24.5 ± 11.1 GPa.

**Figure 3:**
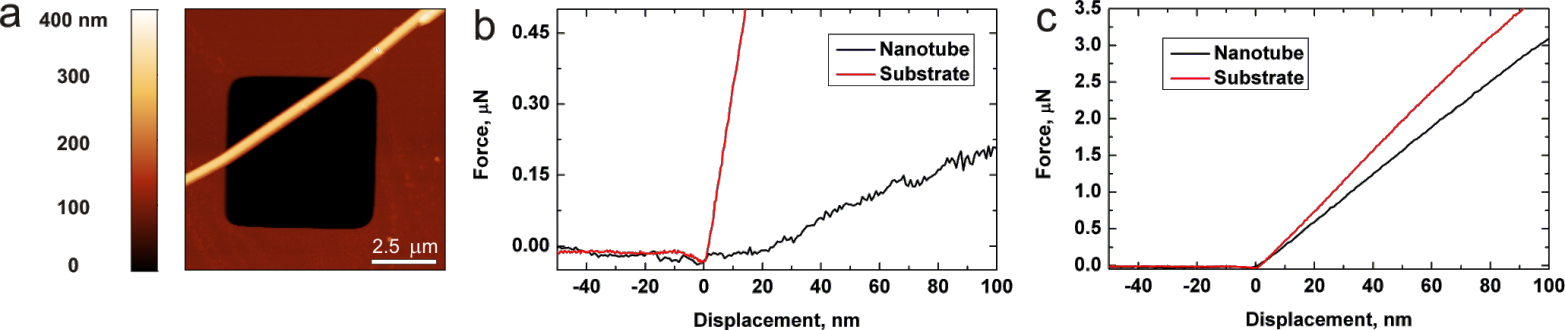
Three-point bending test and nanoindentation. AFM image of suspended silica NT (a); force–distance curve taken on the suspended part of the NT (b); nanoindentation force–distance curve taken on adhered part of the same NT (c).

**Figure 4:**
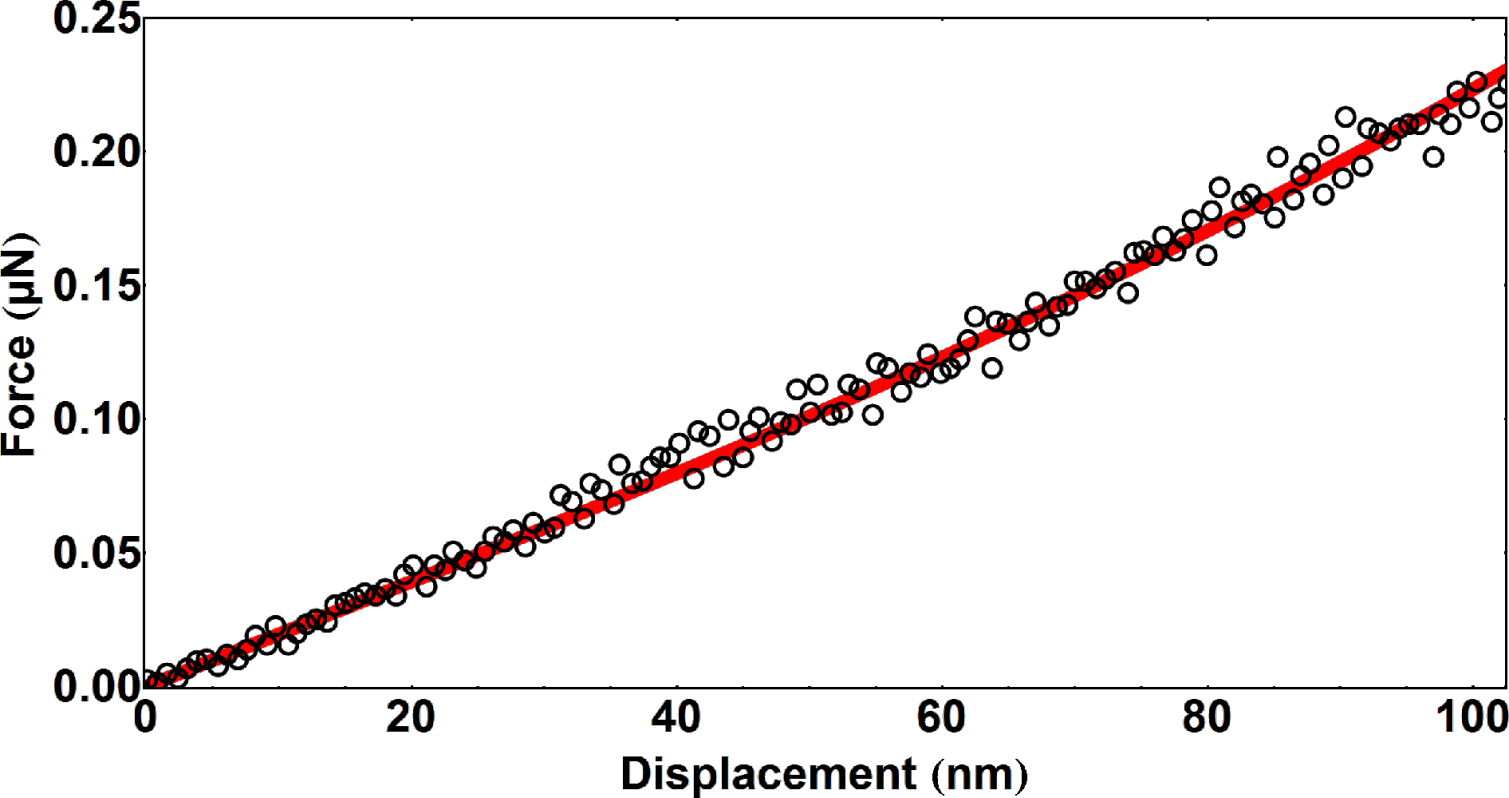
Fitting of three-point bending test of silica NT in AFM.

In AFM measurements the built-in optical microscope was used to find appropriate SiO_2_ NTs for the three-point bending and the nanoindentation tests. Prior to the three-point bending test an AFM image of a NT suspended over a trench was taken in tapping mode at low magnification (typically 10 × 10 μm, Figure 3a). In order to ensure proper tip positioning during force spectroscopy a NT was scanned sequentially at a higher magnification (typically 3 × 3 and 1 × 1 μm). Several force–distance curves were taken in the center of a suspended NT. The curves were linear, and the loading and unloading curves coincide, which indicates an elastic response of the NT. A force–distance curve taken on a hard oxidized silicon substrate is shown for comparison (Figure 3b). Values of Young’s modulus were calculated by using Equation 3 and Equation 4. Fitting results are shown in Figure 4. The initial region, i.e., the region of displacement below the characteristic inner radius of NT, was used for fitting since the high load region would be invalid due to non-linearity and plastic deformation effect. Measurements were performed on five NTs and results are summarized below in Table 1.

Nanoindentation experiments were performed on the same NTs that were used in the three-point bending experiments. A selected area of a NT on the substrate (3 × 3 and 1 × 1 μm) was scanned sequentially prior and after nanoindentation. Several force–distance curves (usually three curves) were taken on a NT with an interval of about 1 μm. Typical force–distance curve shown at Figure 3c. Only the initial linear region was used for analysis.

**Figure 5:**
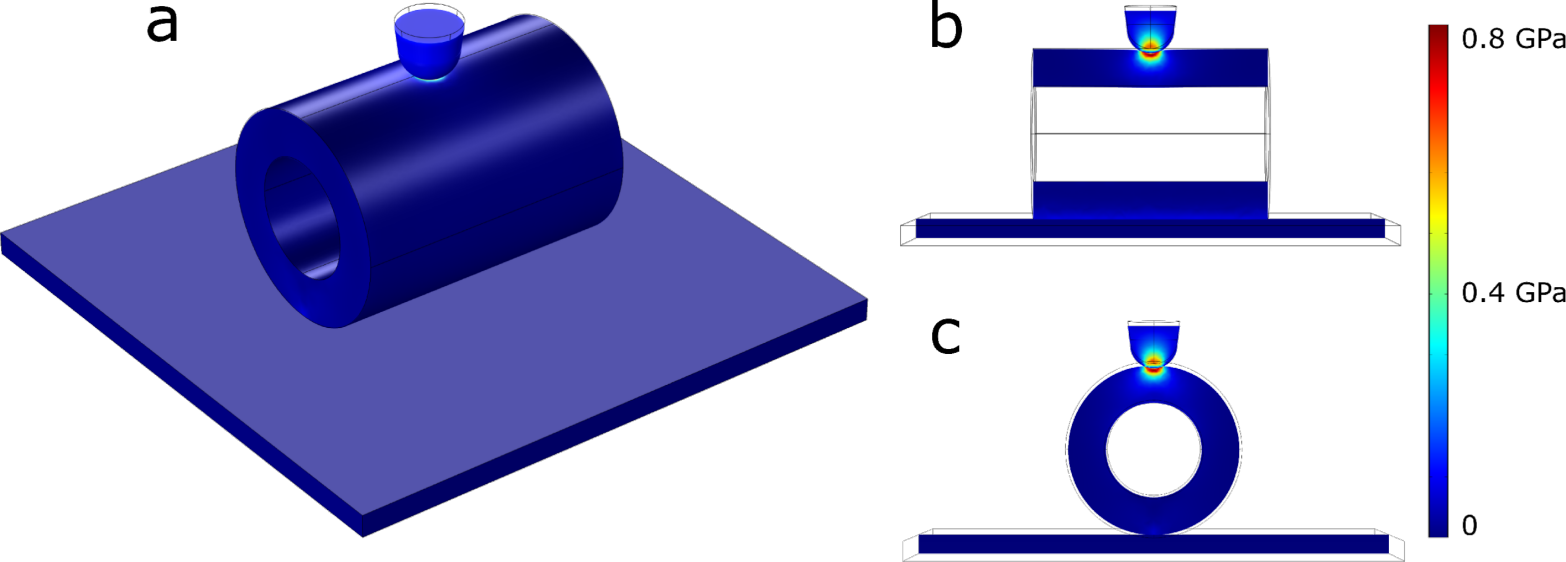
FEM simulation of a SiO_2_ NT nanoindentation. Perspective view (a), longitudinal cross section (b), transversal cross section (c). The colors correspond to the von Mises stress.

The experimental nanoindentation data was processed by using FEM simulations. A constant indentation depth of 5 nm was applied to the shell. This was done due to the fact that in indentation experiments, for small indentation depths, the initial part of the loading curve corresponds to the elastic regime of material response and therefore the experimental values can be compared to the fully elastic result from FEM simulations. A parametric sweep over the Young modulus of the shell was used to calculate the elastic force acting on the indentation tip and was then compared to the experimental force–displacement curve. Geometrical parameters of indenter and each individual shell were determined from HRSEM images (tip radius 25 nm). An example of a FEM simulation of indentation is shown in Figure 5. Additionally, the thin-shell and Hertz models were used for comparison. The thin-shell model allows to neglect the shape of the indenter and consider only the membrane-like deformation of the NT. The Hertz model, on the contrary, describes the indentation of a spherical tip into a flat substrate only and ignores the possible elastic compressing of a NT. According to FEM simulations, in case of nanoindentation of thick-walled SiO_2_ NTs there are both compression and indentation present. Thus, both models underestimate the Young modulus as can be seen from Table 1.

**Table 1:** Young’s moduli of NTs measured by three-point bending and nanoindentation methods by AFM.

nr.	*R*_o_, outer radius, nm	*R*_i_, inner radius, nm	*E*_TPB_, three-point bending, GPa	*E*_shell_, nanoindent., GPa	*E*_hertz_*_,_* nanoindent., GPa	*E*_FEM_, nanoindent., GPa

1	91	56	42.3	6.1	5.4	22.0
2	86	50	37.0	11.6	5.9	29.0
3	87	45	41.8	10.0	5.9	21.5
4	115	62	36.5	6.3	2.6	16.8
5	91	41	48.5	5.6	3.0	11.3

The average values of the Young’ modulus measured by half-suspended beam bending, AFM three-point bending and nanoindentation tests (processed by using FEM simulation) were 24.5 ± 11.1 GPa, 41.3 ± 5.8 GPa and 20.1 ± 7.5 GPa, respectively. Our results are in a good agreement with the ones obtained by Dikin et al. (46.5 GPa, [13]). They applied the resonance method on SiO_2_ NWs (diameter 80–100 nm) inside an SEM to determine the Young’s modulus. However, our values are lower than those obtained by Ni et al. (76.6 GPa for 50–90 nm NWs) and Houmadi (73.3 GPa for 35 nm NTs) by using the three-point bending method [14–15]. The difference in Young’s modulus values of SiO_2_ NTs and NWs can be attributed to the size effect and the parameters of the chemical reaction. It is well-known, that mechanical properties of silica gels strongly depends on post-treatment procedures (aging time and annealing temperature). With an increase of annealing temperature, the density of macroscopic samples of silica gel is increasing as are its hardness and Young’s modulus, approaching values of fused silica at ca. 1000 °C [30]. High values of the Young’s modulus of sol–gel derived silica NTs [15] even without annealing can be explained by the size effect, which facilitates an effective evaporation of chemical reaction residuals and shrinking of NTs.

We would like to note some peculiarities of the methods used in our study for the characterization of SiO_2_ NTs (taking into account the specific properties of this material). The in situ SEM cantilever beam bending method benefits from visual guidance of the bending process, and can be applied to brittle materials or to metals with a well-pronounced elastic-to-plastic transition [24,31]. Since the bending profile of the test object is registered visually by SEM, the smaller the deformation, the higher the error. In SEM, SiO_2_ NTs demonstrated limited elasticity and enhanced plasticity, caused by the e-beam promoted generation of defects and their enhanced mobility as it was demonstrated by Zheng et al. for amorhous silica NPs and NWs [20]. It can lead to softening and plastic deformation of the material at large bending angles. Thus, the in situ SEM cantilever beam bending method can give an underestimated Young’s modulus for SiO_2_ NTs.

The procedure of AFM nanoindentation is rather simple. However, in case of thick-walled nanotubes data processing is complicated and requires FEM simulations and separate HRSEM characterization of the inner and outer diameters. Hertz and membrane models, commonly used for nanoindentation of solid and tubular objects, are inappropriate in case of thick-walled NTs. Moreover, the nanoindentation method is sensitive to local defects and applicable only in the case of a highly homogeneous structure.

Three-point bending test of NTs also suffers from the need of separate electron microscope characterization. However even small displacement of the AFM tip, and thus small deformation of suspended tube, can be measured with high accuracy at small applied force. The experimental data can be easily processed by using simple analytical equations. In our opinion, three-point bending is the most appropriate method for mechanical characterization of thick-walled NTs with limited elasticity.

## Conclusion

In this work we measured the Young’s modulus of SiO_2_ nanotubes by using three different methods. Half-suspended bending tests were carried out inside a SEM by using a nanomanipulator equipped with force sensor. The average value of the Young modulus was found to be 24.5 ± 11.1 GPa. Unexpectedly, significant plasticity was observed. Nanoindentation and three-point bending tests were performed on the same set of NTs under ambient conditions, resulting in values of 20.1 ± 7.5 GPa and 41.3 ± 5.8 GPa, respectively. Three-point bending tests were found to be the most appropriate method for measuring the Young’s modulus of sol–gel synthesized SiO_2_ NTs.

## Supporting Information

File 1 Additional SEM images of broken and collapsed SiO_2_ NTs; additional information on in situ SEM bending; some details on FEM simulations; QTF force sensor calibration procedure.
Additional experimental data.
