# Supplementary material for: Mechanical properties of sol–gel derived SiO2 nanotubes
Source: Beilstein J Nanotechnol. 2014 Oct 20;5:1808–14. doi: 10.3762/bjnano.5.191 (PMC4222284; doi:10.3762/bjnano.5.191)
Supplement: File 1 — Additional SEM images of broken and collapsed SiO2 NTs; additional information on in situ SEM bending; some details on FEM simulations; QTF force sensor calibration procedure. Additional experimental data. [file Beilstein_J_Nanotechnol-05-1808-s001.pdf]

Supporting Information

for

Mechanical properties of sol–gel derived SiO_2 nanotubes

Boris Polyakov*[1], Mikk Antsov[2,3], Sergei Vlassov[1], Leonid M Dorogin[2,3,4], Mikk Vahtrus[2,3], Roberts Zabels[1], Sven Lange[2,3] and Rünno Lõhmus[2,3]

Address: [1]Institute of Solid State Physics, University of Latvia, Kengaraga st. 8, LV-1063, Riga, Latvia, [2]Institute of Physics, University of Tartu, Ravila 14c, 50412, Tartu, Estonia, [3]Estonian Nanotechnology Competence Centre, Ravila 14c, 50412, Tartu, Estonia and [4]ITMO University, Kronverkskiy pr., 49, 197101, Saint Petersburg, Russia

Email: Boris Polyakov* - boriss.polakovs@cfi.lu.lv

* Corresponding author

Additional experimental data

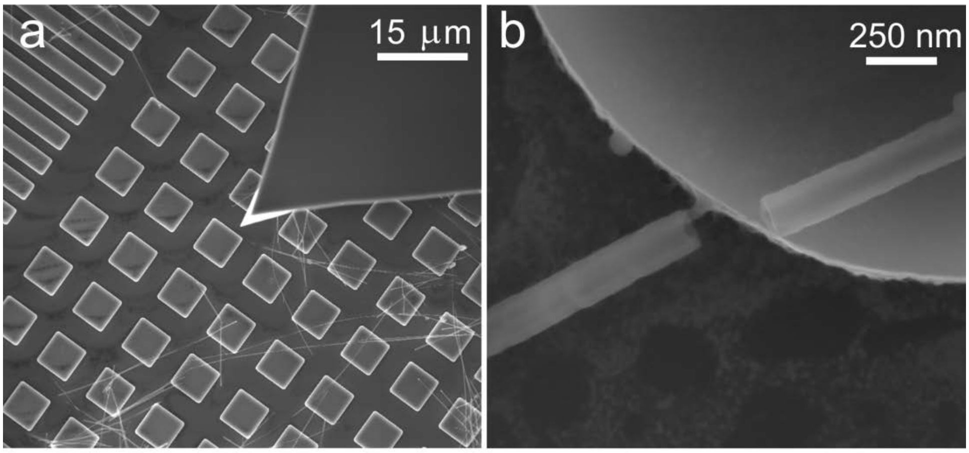

Figure S1: SiO2 NTs on patterned substrate. Half-suspended and bridged NTs are visible (a). Broken NT, inner diameter is visible (b).

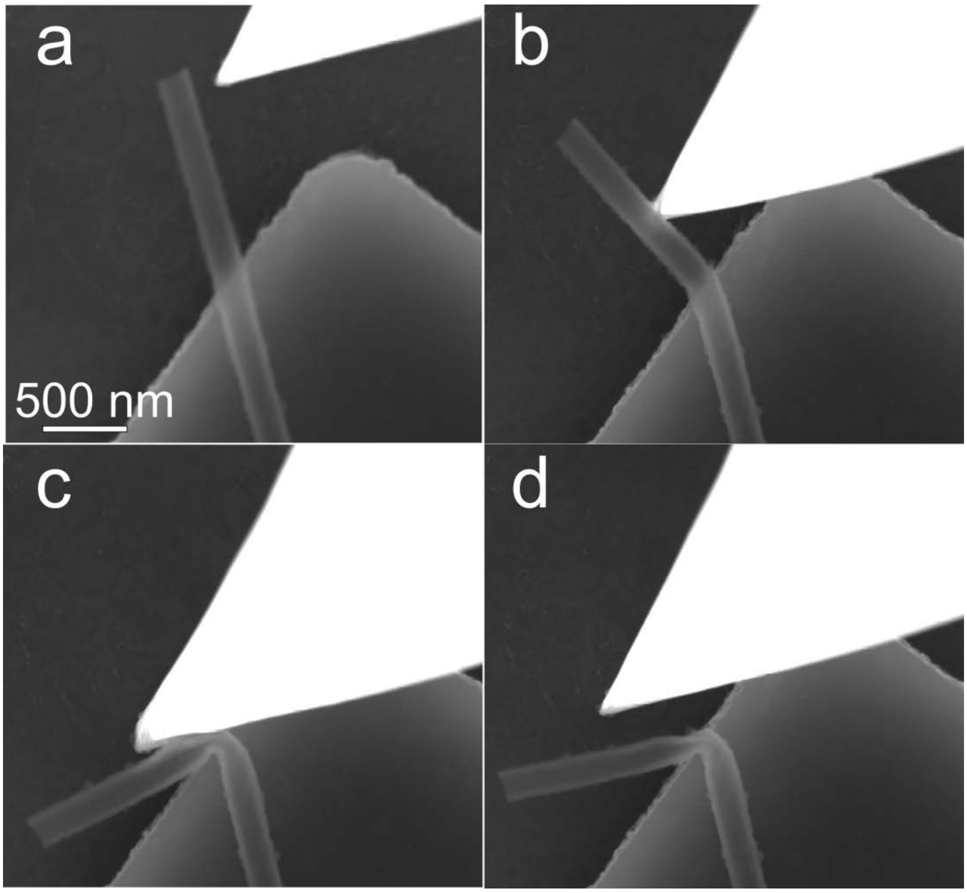

Figure S2: SiO2 NT bending and collapsing.

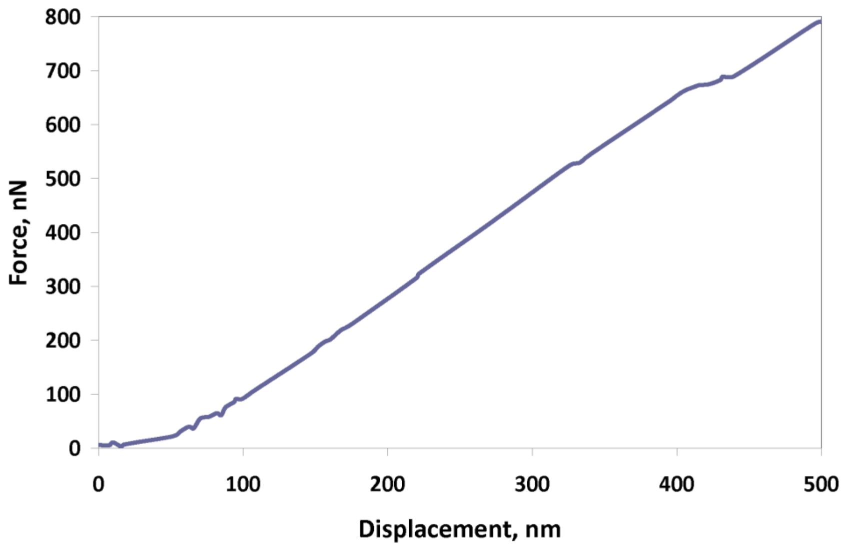

Figure S3: Force–displacement curve of half-suspended NT bending inside SEM.

Table S1: Young's moduli of NTs measured by half-suspended beam bending method inside SEM.

Nr.	R_o, outer radius, nm	R_i, inner radius, nm	E_{hsb}, half-suspended beam, GPa
1	76	32.5	50
2	91	41.5	25
3	75	32.5	25
4	110	55	22
5	113	62.5	15
6	80	32	10
7	65	42.5	20
8	79	32.5	35
9	101	57.5	17
10	92.5	38	35
11	105	57.5	15
12	95	32.5	25

Additional details on FEM

The whole mesh for each simulation consisted of approx. $3 \cdot 10^4$ elements. In order to save computation time the length of the shell was chosen to be approximately 20% of the real length. The difference between the results from the full and partial systems was found to be negligible. It was made denser at the interface between the tip and shell and consisted of elements with average size of 0.25 nm. The SiO_2 substrate was made to consist of elements with average size 0.15 microns.

QTF force sensors calibration procedure

QTF force sensors were calibrated on a reference contact mode AFM cantilever (force constant c = 0.056 Nm) previously calibrated by the thermal noise method. QTF sensor mounted on nanomanipulator was pushed against the reference cantilever using continuous step regime of manipulator as it shown in Figure S4. Force exerted by the reference cantilever on QTF sensor was calculated by multiplication of cantilever displacement and cantilever force constant. QTF amplitude and x-axis displacement signal from the manipulator were recorded simultaneously (Figure S5). However, for conversion of QTF amplitude into force units, force should be plotted as function of QTF amplitude (Figure S6), and equation should be found force = $f \cdot (QTF_{amplitude})$.

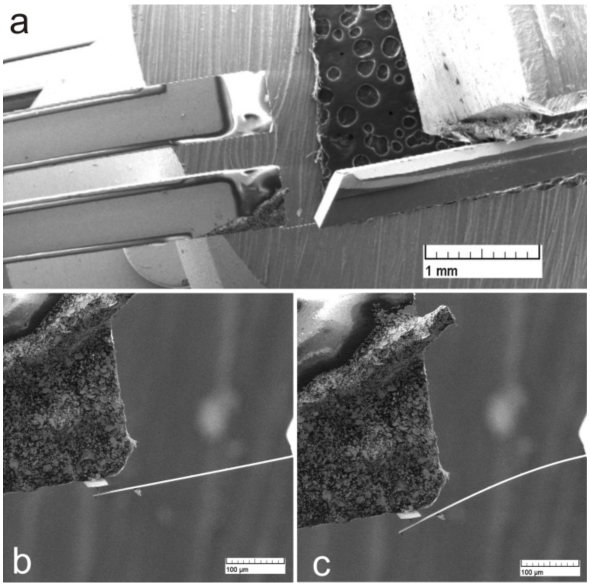

Figure S4: QTF sensor mounted on the nanomanipulator.

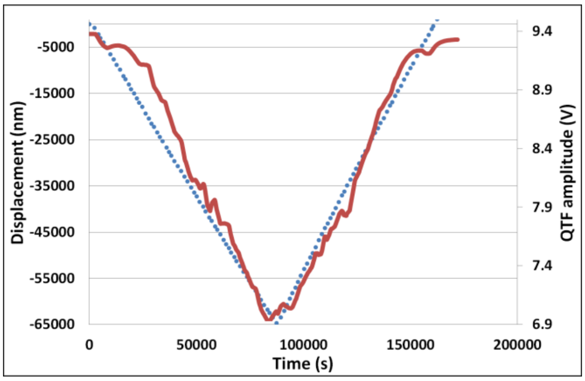

Figure S5: QTF amplitude and *x*-axis displacement signal from the nano-manipulator.

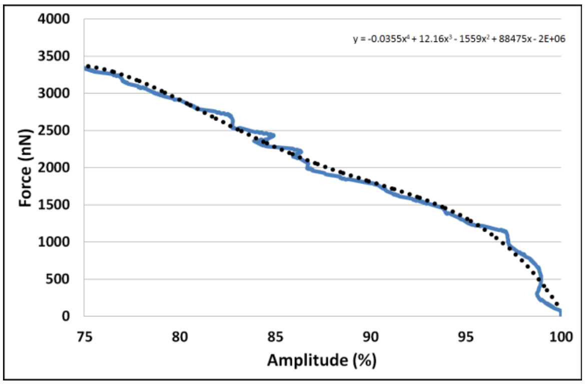

Figure S6: Force exerted on the QTF sensor as function of the amplitude.
